# Hashimoto’s Thyroiditis and Female Infertility: A Clinical and Statistical Investigation of Endocrine and Ovarian Markers

**DOI:** 10.3390/jcm14134770

**Published:** 2025-07-06

**Authors:** Emilia Cristina Popa, Laura Maghiar, Teodor Andrei Maghiar, Ilarie Brihan, Laura Monica Georgescu, Bianca Anamaria Toderaș, Liliana Sachelarie, Loredana Liliana Hurjui, Anca Huniadi

**Affiliations:** 1 Department of Medical Sciences, Faculty of Medicine and Pharmacy, University of Oradea, 1st December Square 10, 410073 Oradea, Romania; emilia.cristinapopa@gmail.com (E.C.P.); lgeorgescu@uoradea.ro (L.M.G.); toderas.biancaanamaria@student.uoradea.ro (B.A.T.); 2 Department of Psychoneurosciences and Recovery, Faculty of Medicine and Pharmacy, University of Oradea, 1st December Square 10, 410073 Oradea, Romania; laura.maghiar@uoradea.ro (L.M.); brihan_drm@yahoo.com (I.B.); 3 Department of Surgery, Faculty of Medicine and Pharmacy, University of Oradea, 1st December Square 10, 410073 Oradea, Romania; teodormaghiar@yahoo.com (T.A.M.); ahuniadi@uoradea.ro (A.H.); 4 Preclinical Sciences Department, Faculty of Medicine, Apollonia University, 700511 Iasi, Romania; 5 Morpho-Functional Sciences II Department, Discipline of Physiology, Faculty of Medicine, “Grigore T. Popa” University of Medicine and Pharmacy Iasi, 16 Universitatii Str., 700115 Iasi, Romania

**Keywords:** Hashimoto’s thyroiditis, AMH, TSH, ovarian reserve, infertility, autoimmune disease, female reproduction

## Abstract

**Background:** Hashimoto’s thyroiditis (HT), the most prevalent autoimmune thyroid disorder in reproductive-age women, has been linked to diminished ovarian reserve and subfertility. This study aimed to evaluate the relationship between HT and key fertility parameters, including hormonal markers and reproductive outcomes, while also exploring the potential impact of thyroid hormone replacement therapy. **Methods:** A retrospective observational study was conducted on 86 women undergoing fertility evaluation. Participants were divided into two groups based on anti-thyroid peroxidase antibodies (ATPO): the HT group (n = 49) and the control group (n = 37). Among women with HT, 57% were receiving levothyroxine (Euthyrox^®^) at the time of assessment. Variables analyzed included serum levels of anti-Müllerian hormone (AMH), thyroid-stimulating hormone (TSH), insulin resistance index (HOMA-IR), number of oocytes retrieved, blastocysts formed, pregnancies achieved, and live births. Statistical methods included *t*-tests, Mann–Whitney U tests, Pearson/Spearman correlations, and linear regression models. **Results:** Women in the HT group had slightly lower AMH levels and oocyte counts compared to controls, though these differences did not reach statistical significance. TSH values were higher in the HT group and showed a significant negative correlation with blastocyst formation (*p* = 0.03). Although TSH also showed negative trends with oocyte count, pregnancies, and live births, these correlations did not reach statistical significance. A post-hoc subgroup analysis revealed that HT patients receiving levothyroxine tended to have higher numbers of oocytes retrieved and blastocysts formed compared to untreated HT patients, suggesting a possible beneficial effect of thyroid hormone replacement, although the differences were not statistically significant. **Conclusions:** HT is associated with subtle but clinically relevant impairments in ovarian reserve and reproductive potential. Thyroid hormone replacement may offer modest benefits and should be considered in the individualized management of fertility in women with thyroid autoimmunity.

## 1. Introduction

Hashimoto’s thyroiditis (HT) represents the most common autoimmune thyroid disorder affecting women of reproductive age, with a reported prevalence ranging from 5% to 15%, depending on demographic and geographic factors [1]. This condition is immunologically defined by the presence of circulating anti-thyroid peroxidase antibodies (ATPO), which initiate a gradual autoimmune-mediated destruction of thyroid tissue, ultimately leading to thyroid dysfunction and clinical hypothyroidism. Beyond its endocrine implications, HT has significant repercussions on female reproductive health, as thyroid hormones are critically involved in regulating key reproductive processes such as folliculogenesis oocyte maturation, ovulation, luteal phase integrity, and endometrial receptivity, making even subclinical alterations potentially disruptive to fertility and implantation [2,3]. Growing evidence has increasingly supported a link between thyroid dysfunction—both overt and subclinical—and various aspects of female reproductive impairment, including a measurable decline in ovarian reserve, disruptions in normal ovulatory function, and reduced fertility potential. Moreover, thyroid autoimmunity has been associated with poorer outcomes in assisted reproductive technologies (ART), such as lower oocyte yield, compromised embryo quality, reduced implantation rates, and increased risk of early pregnancy loss [1,2,3,4]. These findings underscore that even subclinical disturbances in thyroid homeostasis may exert a disproportionately significant influence on the delicate endocrine–reproductive axis, warranting their systematic inclusion in the diagnostic and therapeutic algorithms applied to women undergoing fertility evaluation and treatment [3,4]. Serum anti-Müllerian hormone (AMH), widely recognized as a reliable biomarker of ovarian reserve, has been consistently reported to be lower in women with autoimmune thyroid disease, including those who are clinically euthyroid. This suggests that thyroid autoimmunity may negatively impact ovarian follicular dynamics and the functional pool of antral follicles, even without overt thyroid hormone abnormalities. Such findings underscore a possible subclinical disruption in the ovarian environment driven by immune-mediated mechanisms, which may precede detectable changes in TSH or free hormone levels [4,5,6,7]. Additionally, elevated thyroid-stimulating hormone (TSH) levels, frequently observed in HT, may interfere with ovarian responsiveness and embryo implantation [8,9,10].

While multiple studies support a potential association between Hashimoto’s thyroiditis (HT) and impaired female fertility, the overall body of literature remains inconsistent and, at times, contradictory. Some investigations have failed to demonstrate significant differences in anti-Müllerian hormone (AMH) levels, oocyte yield, or pregnancy outcomes between women with autoimmune thyroid disease and those without, particularly in cases where thyroid function remained within normal limits. These discrepancies may be attributed to variations in study design, sample size, population characteristics, and the extent of thyroid autoimmunity. This highlights the need for more standardized, controlled, and longitudinal research to elucidate the actual impact of HT on reproductive potential [6,7]. In contrast, others reported impaired oocyte quality, increased miscarriage rates, and poorer IVF outcomes in the presence of thyroid autoimmunity [8,9,10,11,12,13,14].

This study aims to comprehensively explore the relationship between Hashimoto’s thyroiditis and female fertility by assessing ovarian reserve through anti-Müllerian hormone (AMH) levels, investigating the association between thyroid-stimulating hormone (TSH) and key reproductive parameters, and comparing pregnancy outcomes including previous in vitro fertilization (IVF) history between women with and without thyroid autoimmunity.

Our analysis highlighted potential patterns, such as a negative correlation between TSH and blastocyst formation, which warrant further investigation in larger, prospective studies. Through the inclusion of detailed hormonal profiling including anti-Müllerian hormone (AMH) as a marker of ovarian reserve, thyroid-stimulating hormone (TSH) to assess thyroid axis activity, and the homeostatic model assessment of insulin resistance (HOMA-IR) to capture underlying metabolic dysfunction, this study offers a multidimensional view of the endocrine landscape in these patients. Additionally, by incorporating reproductive history and objective fertility parameters such as the number of oocytes retrieved, blastocyst development, pregnancies, and live births, the analysis moves beyond isolated biochemical findings to a more holistic assessment of reproductive potential. This comprehensive approach provides new insights into the interplay between autoimmune thyroid dysfunction and fertility outcomes, highlighting how subtle endocrine and metabolic disturbances may converge to influence female reproductive health, even in the absence of overt hypothyroidism.

This study provides new insights into the complex relationship between thyroid autoimmunity and female reproductive health, with implications for more personalized fertility management.

## 2. Materials and Methods

### 2.1. Study Design and Participants

This retrospective observational study was conducted on a cohort of 86 women who presented for fertility assessment between 1 January 2024 and 30 December 2024. All participants were evaluated at a reproductive endocrinology clinic and provided informed consent to use their anonymized clinical data. This study was conducted following the Declaration of Helsinki and approved by the ethical Committee of Calla Center, under reference number 1011/A from 28 December 2023. Eligible participants were women of reproductive age (18–45 years) with available thyroid and fertility-related laboratory data. Patients with incomplete hormonal profiles, a history of thyroidectomy, or current thyroid hormone replacement not related to autoimmune thyroiditis were excluded.

Among the 49 women in the Hashimoto group, 28 (57%) were under levothyroxine (Euthyrox^®^, Darmstadt, Hesse, Germany) therapy at the time of evaluation. Interestingly, six women (16%) in the ATPO-negative group were also receiving Euthyrox, possibly for subclinical hypothyroidism or other non-autoimmune indications.

### 2.2. Group Allocation

Participants were divided into two distinct groups according to their thyroid autoimmunity status: the Hashimoto group (HT group), comprising women who tested positive for anti-thyroid peroxidase antibodies (ATPO+), and the control group, consisting of women with negative ATPO results and no clinical or biochemical signs of thyroid dysfunction. The final analysis included 86 women, 49 of whom were assigned to the Hashimoto group (ATPO-positive) and 37 to the control group (ATPO-negative).

### 2.3. Variables and Data Collection

A set of clinical and laboratory variables was collected for each participant enrolled in this study to ensure a multidimensional evaluation of endocrine, metabolic, and reproductive health. Thyroid function was assessed through both qualitative and quantitative markers, specifically the presence of anti-thyroid peroxidase antibodies (ATPO, qualitative) as an indicator of autoimmune thyroid status, and serum thyroid-stimulating hormone (TSH, expressed in mIU/L), which reflects the functional state of the hypothalamic–pituitary–thyroid axis. Evaluation of ovarian reserve and metabolic function included serum anti-Müllerian hormone (AMH, ng/mL), a key marker for assessing the quantity of remaining follicles; the homeostatic model assessment of insulin resistance (HOMA-IR), calculated using fasting glucose and insulin concentrations, to estimate metabolic disturbance; and total testosterone (ng/mL), to assess androgen status.

All blood samples were collected via venipuncture after overnight fasting (minimum 8 h) on days 2–4 of the menstrual cycle. Serum anti-Müllerian hormone (AMH) levels were measured using a second-generation ELISA kit (Beckman Coulter AMH Gen II, Beckman Coulter, Brea, CA, USA), with intra-assay and inter-assay coefficients of variation below 8%. Thyroid-stimulating hormone (TSH) and total testosterone were assessed using chemiluminescence immunoassays (CLIA) on a Cobas e411 analyzer (Roche Diagnostics, Roche Holding AG, Basel, Switzerland). Fasting glucose and insulin were measured via enzymatic and immunometric assays, respectively, and the HOMA-IR index was calculated using the standard formula. All samples were performed at the same certified laboratory under standardized conditions. In addition to endocrine profiling, fertility-related outcomes were meticulously documented. These included the number of oocytes retrieved during stimulation cycles, the number of blastocysts formed, the total number of pregnancies achieved, the number of live births, and any prior history of assisted reproductive technology (ART), particularly in vitro fertilization (IVF). To account for potential confounders in reproductive performance, anthropometric data were also collected for all participants, including patient age (in years) and body mass index (BMI, kg/m^2^). All participants provided written informed consent for using their anonymized clinical and laboratory data in this research. This study was conducted according to the ethical standards of the institutional research committee and adhered to the principles outlined in the Declaration of Helsinki.

### 2.4. Statistical Analysis

Statistical analysis was conducted using IBM SPSS Statistics version 26.0 (IBM Corp., Armonk, NY, USA). The normality of continuous variables was evaluated using the Shapiro–Wilk test. For group comparisons, independent samples *t*-tests were applied to normally distributed variables, while the Mann–Whitney U test was used when the distribution was non-normal.

Pearson correlation coefficients were computed to investigate the relationships between hormonal/metabolic markers (such as TSH, AMH, HOMA-IR, and testosterone) and reproductive outcomes (including the number of oocytes, blastocysts, pregnancies, and live births). Where appropriate, Spearman correlations were used for variables that did not meet the assumptions of normality.

In addition, a simple linear regression model was employed to explore the independent effects of age and Hashimoto’s thyroiditis status on AMH levels. After adjusting for maternal age, this allowed for a more precise assessment of whether autoimmune thyroid disease contributes to diminished ovarian reserve.

## 3. Results

### 3.1. Baseline Characteristics

The study participants’ baseline characteristics are presented according to thyroid autoimmunity status. Table 1 shows 49 women in the Hashimoto group (ATPO-positive) and 37 in the control group (ATPO-negative).

Women in the Hashimoto group were significantly older than those in the control group (mean age 37.6 vs. 33.6 years, *p* = 0.0058). Although the mean AMH level was lower in the Hashimoto group (1.96 vs. 2.35 ng/mL), the difference was insignificant (*p* = 0.2408). Similarly, despite a trend toward higher values in the autoimmune group, no significant differences were observed for TSH, HOMA-IR, or BMI between groups (*p* > 0.05). These findings suggest that age is the only baseline variable significantly different between groups.

### 3.2. Reproductive and Fertility Outcomes

As shown in Table 2, the cohort’s overall reproductive performance was modest, with a mean of approximately 10 oocytes retrieved, two blastocysts formed, and less than one pregnancy and live birth per patient. This reflects significant variability and the complex reproductive profiles typical of women undergoing fertility assessment.

### 3.3. Comparison of Hormonal and Clinical Parameters

As shown in Table 3, no statistically significant differences were observed between the Hashimoto and control groups regarding the number of oocytes retrieved, blastocysts formed, pregnancies achieved, or live births. These findings suggest that the presence of Hashimoto’s thyroiditis does not negatively impact IVF outcomes under appropriate clinical management.

Although all reproductive parameters, namely the number of oocytes retrieved, blastocysts formed, pregnancies, and live births, were lower in the Hashimoto group compared to controls, none of these differences reached statistical significance (*p* > 0.05), indicating that the observed trends may reflect variability rather than an actual effect of thyroid autoimmunity.

### 3.4. Age and Anthropometric Difference

Table 4 compares groups related to age and BMI.

In Table 4, women in the Hashimoto group were significantly older than those in the control group (37.6 ± 4.6 vs. 33.6 ± 6.6 years, *p* = 0.0058). Although BMI was higher in the Hashimoto group (31.6 ± 6.0 vs. 27.4 ± 3.4 kg/m^2^), this difference did not reach statistical significance (*p* = 0.3929). These findings suggest that age, rather than body composition, may play a more prominent role in the reproductive profile of women with thyroid autoimmunity.

### 3.5. Correlations Between Endocrine, Metabolic, and Demographic Predictors and Fertility Parameters

The analysis revealed weak to moderate correlations between thyroid, metabolic, and demographic predictors and various fertility outcomes. The strongest association was observed between TSH and the number of oocytes retrieved (r = −0.412, *p* = 0.089), suggesting that thyroid dysfunction may indirectly affect ovarian response (Table 5).

Although TSH values were higher in the HT group, the correlation with AMH was weakly positive and not statistically significant (r = 0.064, *p* = 0.802).

Among all predictors, age showed the strongest inverse correlation with AMH (r = −0.357), reinforcing its well-established role in ovarian reserve decline. However, all *p*-values were above 0.05, indicating that none of these associations reached statistical significance in this sample.

### 3.6. Correlation and Analytical Insights

This comprehensive table summarizes all observed correlations. While no association reached conventional statistical significance (*p* < 0.05), notable trends such as the negative correlation between TSH and the number of oocytes, and the inverse association between age and AMH, suggest potential physiological links that warrant further exploration in larger cohorts, Table 6.

### 3.7. Comparative Analysis Between Groups (ATPO-Positive vs. ATPO-Negative)

The results revealed a statistically significant age difference, with women in the ATPO-positive group being significantly older on average (*p* = 0.002). No statistically significant differences were observed for all other variables, including AMH and the number of oocytes (*p* > 0.05), although there were clinically notable trends favoring the control group (Table 7).

### 3.8. Comparative Analysis Within the HT Group: Treated vs. Untreated Patients

Among the 49 women in the Hashimoto thyroiditis (HT) group, 28 (57%) were receiving levothyroxine (Euthyrox^®^) therapy, while 21 (43%) were untreated at the time of fertility assessment. A comparative analysis was performed to explore potential differences in hormonal and reproductive parameters between these subgroups.

As shown in Table 8, treated women had slightly higher mean values for AMH, number of oocytes retrieved, blastocysts formed, and clinical pregnancies compared to untreated women. However, none of these differences reached statistical significance (*p* > 0.05). These trends suggest a potential beneficial effect of thyroid hormone replacement on ovarian response and early reproductive outcomes in women with autoimmune thyroiditis. Nonetheless, due to the limited sample size and retrospective nature of this study, these findings should be interpreted with caution and require confirmation in larger prospective cohorts.

## 4. Discussion

This study contributes to the growing body of literature exploring the interplay between autoimmune thyroiditis and female reproductive health, focusing on ovarian reserve and fertility outcomes. Although not all observed differences reached statistical significance, the overall pattern of results supports the hypothesis that Hashimoto’s thyroiditis (HT) may exert a subtle but clinically relevant impact on reproductive function.

A key finding from our cohort was the significantly higher mean age of women in the HT group compared to controls (37.6 vs. 33.6 years, *p* = 0.002). This age difference may partially explain the trend toward diminished ovarian reserve in the autoimmune group. Age remains the strongest independent predictor of ovarian function, including AMH levels, oocyte yield, and pregnancy potential [14]. Indeed, our analysis confirmed a negative correlation between age and AMH (r = −0.357) and between age and the number of oocytes and pregnancies. These findings are biologically plausible and well-documented in prior studies [1,3,14].

While the mean AMH level was lower in women with HT (1.96 vs. 2.35 ng/mL), this difference did not reach statistical significance (*p* = 0.24). Similarly, the number of retrieved oocytes, blastocysts, pregnancies, and live births was lower in the Hashimoto group, but none of these outcomes differed significantly between groups. These results are consistent with other studies that report a trend toward reduced ovarian reserve and fertility indicators in women with thyroid autoimmunity, even in the absence of overt hypothyroidism [5,6,7,15,16,17,18,19,20]. The lack of significance may be attributable to the relatively small sample size and this study’s retrospective nature.

More nuanced insights were revealed through the correlation analysis. In the ATPO-positive group, we observed a moderate and statistically significant negative correlation between age and AMH (r = −0.49, *p* < 0.01), as well as between age and the number of oocytes retrieved (r = −0.54, *p* < 0.01). Additionally, a positive and significant correlation was found between the number of oocytes and blastocysts (r = 0.59, *p* = 0.002), supporting that reduced follicular output directly impacts embryo development potential. These internal patterns persisted even when mean values between groups did not differ significantly, illustrating the importance of age-related decline in ovarian performance among women with thyroid autoimmunity.

Although falling within the euthyroid range, TSH levels showed a statistically significant negative correlation with the number of blastocysts (r = −0.513, *p* = 0.03). A similar but non-significant trend was noted for the correlation between TSH and the number of oocytes (r = −0.412, *p* = 0.089). These findings align with prior reports suggesting that even mild elevations in TSH within the high–normal range may affect oocyte quality and embryo development, particularly in patients undergoing assisted reproduction [12,13,21,22]. Current clinical guidelines often recommend maintaining TSH < 2.5 mIU/L in fertility protocols, and our results further support this recommendation.

Concerning metabolic markers, HOMA-IR and serum testosterone exhibited weak, non-significant negative correlations with AMH and reproductive outcomes. Although these associations were not statistically significant, they suggest a possible multifactorial mechanism involving insulin resistance and androgen imbalance, which may contribute to the subfertility observed in HT patients [23,24,25,26].

This study presents a descriptive evaluation of hormonal, metabolic, and reproductive markers in a well-characterized cohort of women undergoing fertility assessment. Our analysis highlighted potential patterns, such as a negative correlation between TSH and blastocyst formation, which may warrant further investigation in larger, prospective studies. Comparable findings were recently published by Sušanj Šepić et al., who demonstrated impaired oocyte maturation and embryonic development in euthyroid women with thyroid autoimmunity undergoing ART [15]. Moreover, metabolomic analyses, such as those of da Silva Bastos et al., support the hypothesis that the follicular microenvironment in HT patients is altered at a biochemical level, even when standard hormonal markers appear normal [27].

Although our results align with earlier studies showing lower AMH levels and compromised ovarian reserve in women with HT [6,7], they also underscore the importance of interpreting reproductive outcomes in a broader clinical context. Autoimmune thyroid disease does not universally impair fertility but may act synergistically with age and metabolic factors to reduce reproductive potential.

In our cohort, 57% of ATPO-positive women received levothyroxine (Euthyrox^®^) therapy, and their reproductive outcomes, including oocyte yield, blastocyst development, and live births, were slightly improved compared to those not receiving treatment. This observation supports the hypothesis that thyroid hormone supplementation may mitigate some reproductive impairments associated with autoimmune thyroiditis. A similar trend was noted in the ATPO-negative group, where a small subset of Euthyrox-treated women achieved higher pregnancy and live birth rates despite lower follicular output, suggesting potential benefits even in subclinical cases.

A post-hoc subgroup analysis within the HT group compared treated and untreated women. Although the differences did not reach statistical significance, women receiving levothyroxine therapy exhibited a trend toward higher AMH levels, greater oocyte yield, and increased blastocyst formation. These findings may indicate a beneficial effect of thyroid hormone replacement on ovarian function and fertility, even in euthyroid women with thyroid autoimmunity. This observation is consistent with previous reports suggesting that levothyroxine treatment may improve assisted reproduction outcomes in women with subclinical hypothyroidism or thyroid autoimmunity [28,29,30].

Although the retrospective design and modest sample size may limit the statistical strength of our conclusions, this study provides clinically relevant insights into the relationship between Hashimoto’s thyroiditis and fertility outcomes, highlighting the potential role of levothyroxine therapy, an observation that reflects real-world practice and deserves confirmation through larger, prospective studies. From a clinical perspective, these findings emphasize the need for early screening of thyroid autoimmunity, even in asymptomatic women undergoing fertility evaluation. Monitoring TSH, AMH, and metabolic parameters like HOMA-IR can help identify women at increased risk of suboptimal ovarian response. A more personalized approach that integrates endocrine, metabolic, and reproductive factors may enhance fertility outcomes in women with Hashimoto’s thyroiditis.

## 5. Conclusions

Hashimoto’s thyroiditis was associated with subtle alterations in ovarian reserve and reproductive parameters, although most differences did not reach statistical significance. A significant negative correlation between TSH and blastocyst formation suggests a possible impact of thyroid function on fertility, even in euthyroid women. Age remained the strongest predictor of ovarian reserve. These findings support the importance of early thyroid screening and individualized fertility management in women with autoimmune thyroid disease. 

## Figures and Tables

**Table 1:** Baseline comparison between the Hashimoto and control groups.

Variable	Hashimoto Mean	Control Mean	*p*-Value
Age (years)	37.63	33.56	0.0058
BMI (kg/m^2^)	31.55	27.39	0.3929
AMH (ng/mL)	1.96	2.35	0.2408
TSH (mIU/L)	2.22	2.11	0.7816
HOMA-IR	2.35	2.34	0.7671

Reference values for AMH vary with age; 1–4 ng/mL is considered functional for reproductive-age women; HOMA-IR > 2.5 may indicate insulin resistance; clinical interpretation depends on patient context.

**Table 2:** Descriptive statistics of reproductive outcomes in the entire cohort.

Parameter	Mean	SD	Median	Min	Max	Count
Number of Oocytes	9.89	7.08	8.0	0.0	34.0	81.0
Number of Blastocysts	2.26	2.12	2.0	0.0	12.0	77.0
Number of Pregnancies	0.78	1.13	1.0	0.0	8.0	76.0
Number of Live Births	0.34	0.58	0.0	0.0	3.0	58.0

**Table 3:** Comparison of hormonal and clinical parameters.

Parameter	Hashimoto Group Mean	Control Group Mean	*p*-Value
Number of Oocytes	9.05	10.89	0.2519
Number of Blastocysts	2.29	2.22	0.8857
Number of Pregnancies	0.85	0.69	0.5047
Number of Live Births	0.41	0.27	0.3564

**Table 4:** Age and BMI: comparison between groups (with *p*-values).

Parameter	Hashimoto Group (Mean ± SD)	Control Group (Mean ± SD)	*p*-Value
Age (years)	37.63 ± 4.63	33.56 ± 6.57	0.0058
BMI (kg/m^2^)	31.55 ± 5.99	27.39 ± 3.38	0.3929

**Table 5:** Correlation between age, TSH, HOMA-IR, testosterone, and fertility parameters.

Predictor	Fertility Marker	Correlation (r)	*p*-Value
TSH	AMH	0.064	0.802
HOMA-IR	AMH	−0.099	0.695
Testosterone	AMH	−0.194	0.441
Age	AMH	−0.357	0.146
TSH	Number of Oocytes	−0.412	0.089
HOMA-IR	Number of Oocytes	−0.284	0.253
TSH	Number of Blastocysts	−0.513	0.030
HOMA-IR	Number of Blastocysts	−0.183	0.467
TSH	Number of Pregnancies	−0.308	0.229
HOMA-IR	Number of Pregnancies	−0.089	0.735
TSH	Number of Live Births	−0.409	0.147
HOMA-IR	Number of Live Births	−0.001	0.997

**Table 6:** Correlation and analytical insights.

Predictor	Fertility Marker	Correlation	*p*-Value
TSH	AMH	0.064	0.802
HOMA-IR	AMH	−0.099	0.695
Testosteron	AMH	−0.194	0.441
Age	AMH	−0.357	0.146
TSH	Number of Oocytes	−0.412	0.089
HOMA-IR	Number of Oocytes	−0.284	0.253
Testosteron	Number of Oocytes	−0.168	0.504
VARSTA	Number of Oocytes	−0.506	0.032
TSH	Number of Blastocysts	−0.513	0.030
HOMA-IR	Number of Blastocysts	−0.183	0.467
Testosteron	Number of Blastocysts	−0.048	0.851
VARSTA	Number of Blastocysts	−0.292	0.239
TSH	Number of Pregnancies	−0.308	0.229
HOMA-IR	Number of Pregnancies	−0.089	0.735
Testosteron	Number of Pregnancies	−0.119	0.649
Age	Number of Pregnancies	−0.519	0.033
TSH	Number of Live Births	−0.409	0.147
HOMA-IR	Number of Live Births	−0.001	0.997
Testosteron	Number of Live Births	−0.099	0.736
Age	Number of Live Births	−0.562	0.037

**Table 7:** Comparative analysis.

Variable	Media (ATPO+)	Media (ATPO−)	*p*-Value
Age	37.63	33.56	0.0023
AMH	1.96	2.54	0.2815
Number of Oocytes	9.05	10.89	0.2519
Number of Blastocysts	2.29	2.22	0.8857
Number of Pregnancies	0.85	0.69	0.5047
Number of Live Births	0.41	0.27	0.3564
TSH	2.22	2.11	0.7278
HOMA-IR	2.35	2.34	0.9804
BMI	31.55	27.39	0.3558

**Table 8:** Comparative analysis of hormonal and reproductive parameters between treated and untreated women with Hashimoto’s thyroiditis (HT).

Parameter	Treated HT (n = 28)	Untreated HT (n = 21)	*p*-Value
Age (years)	37.8 ± 4.2	37.3 ± 5.1	0.654
AMH (ng/mL)	2.05 ± 1.34	1.82 ± 1.26	0.482
Number of oocytes	9.78 ± 7.12	8.11 ± 6.83	0.366
Number of blastocysts	2.50 ± 2.04	1.90 ± 2.16	0.273
Number of pregnancies	0.93 ± 1.22	0.71 ± 1.06	0.534
Number of live births	0.39 ± 0.61	0.43 ± 0.57	0.817
TSH (mIU/L)	2.09 ± 0.96	2.40 ± 1.11	0.221
HOMA-IR	2.29 ± 1.45	2.43 ± 1.52	0.693

## Data Availability

The original contributions presented in this study are included in this article. Further inquiries can be directed to the corresponding authors.
